# Highly sensitive detection of antimicrobial resistance genes in hospital wastewater using the multiplex hybrid capture target enrichment

**DOI:** 10.1128/msphere.00100-23

**Published:** 2023-05-24

**Authors:** Hiroaki Baba, Makoto Kuroda, Tsuyoshi Sekizuka, Hajime Kanamori

**Affiliations:** 1 Department of Infectious Diseases, Internal Medicine, Tohoku University Graduate School of Medicine, Sendai, Miyagi, Japan; 2 Pathogen Genomics Center, National Institute of Infectious Diseases, Tokyo, Japan; JMI Laboratories, North Liberty, Iowa, USA

**Keywords:** antimicrobial resistance, antibiotic resistant genes, metagenomics, water environment, hospital wastewater, one health

## Abstract

Wastewater can be useful in monitoring the spread of antimicrobial resistance (AMR) within a hospital. The abundance of antibiotic resistance genes (ARGs) in hospital effluent was assessed using metagenomic sequencing (mDNA-seq) and hybrid capture (xHYB). mDNA-seq analysis and subsequent xHYB targeted enrichment were conducted on two effluent samples per month from November 2018 to May 2021. Reads per kilobase per million (RPKM) values were calculated for all 1,272 ARGs in the constructed database. The monthly numbers of patients with presumed extended-spectrum β-lactamase (ESBL)-producing and metallo-β-lactamase (MBL)-producing bacteria, methicillin-resistant *Staphylococcus aureus* (MRSA), and vancomycin-resistant enterococci (VRE) were compared with the monthly RPKM values of *bla*_CTX-M_, *bla*_IMP_, *mecA*, *vanA*, and *vanB* by xHYB. The average RPKM value for all ARGs detected by xHYB was significantly higher than that of mDNA-seq (665, 225, and 328, respectively, and *P* < 0.05). The average number of patients with ESBL producers and RPKM values of *bla*_CTX-M-1_ genes in 2020 were significantly higher than that in 2019 (17 and 13 patients per month and 921 vs 232 per month, respectively, both *P* < 0.05). The average numbers of patients with MBL-producers, MRSA, and VRE were 1, 28, and 0 per month, respectively, while the average RPKM values of *bla*_IMP_, *mecA*, *vanA*, and *vanB* were 6,163, 6, 0, and 126 per month, respectively. Monitoring ARGs in hospital effluent using xHYB was found to be more useful than conventional mDNA-seq in detecting ARGs including *bla*_CTX-M_, *bla*_IMP,_ and *vanB*, which are important for infection control.

**IMPORTANCE:**

Environmental ARGs play a crucial role in the emergence and spread of AMR that constitutes a significant global health threat. One major source of ARGs is effluent from healthcare facilities, where patients are frequently administered antimicrobials. Culture-independent methods, including metagenomics, can detect environmental ARGs carried by non-culturable bacteria and extracellular ARGs. mDNA-seq is one of the most comprehensive methods for environmental ARG surveillance; however, its sensitivity is insufficient for wastewater surveillance. This study demonstrates that xHYB appropriately monitors ARGs in hospital effluent for sensitive identification of nosocomial AMR dissemination. Correlations were observed between the numbers of inpatients with antibiotic-resistant bacteria and the ARG RPKM values in hospital effluent over time. ARG surveillance in hospital effluent using the highly sensitive and specific xHYB method could improve our understanding of the emergence and spread of AMR within a hospital.

## INTRODUCTION

The emergence and spread of antimicrobial resistance (AMR), making infections difficult or impossible to treat, constitutes a significant global health threat (1). Since antibiotic-resistant bacteria (ARB) and antibiotic resistance genes (ARGs) circulate through humans and environments, integrated surveillance is necessary to comprehensively understand AMR dynamics in the concept of “One health,” unifying the health of humans, animals, and the environment (2).

Due to the presence of clinically important ARB and corresponding ARGs, including extended-spectrum β-lactamases (ESBLs) and carbapenemases, human wastewater is a significant AMR reservoir and a potential source of river water contamination (1). One major source of ARB and ARGs is effluent from healthcare facilities, where patients are frequently administered antimicrobials (1). As hospital effluent contains excreta (an indicator of inpatient ARB carriage) (3), it can be used to monitor the spread of AMR in a hospital (3).

Environmental ARGs play a crucial role in the emergence and spread of AMR since they can be transmitted between species via mobile genetic elements, like plasmids (1). Culture-independent methods, including metagenomics, can detect environmental ARGs carried by non-culturable bacteria and extracellular ARGs (1). Metagenomic sequencing (mDNA-seq) is one of the most comprehensive methods for environmental ARG surveillance; however, its sensitivity is insufficient for wastewater surveillance (4). Recently, a target enrichment strategy using hybrid capture (xHYB) followed by next-generation sequencing was developed to detect multiple targeted genes in complex metagenomic samples with high sensitivity (5). This study quantitatively assessed ARG abundance in effluent from a university hospital over time using mDNA-seq and the xHYB method and compared the results to the number of hospitalized patients with ARB during the same period.

## RESULTS

### ARGs in hospital effluent

**Fig 1:**
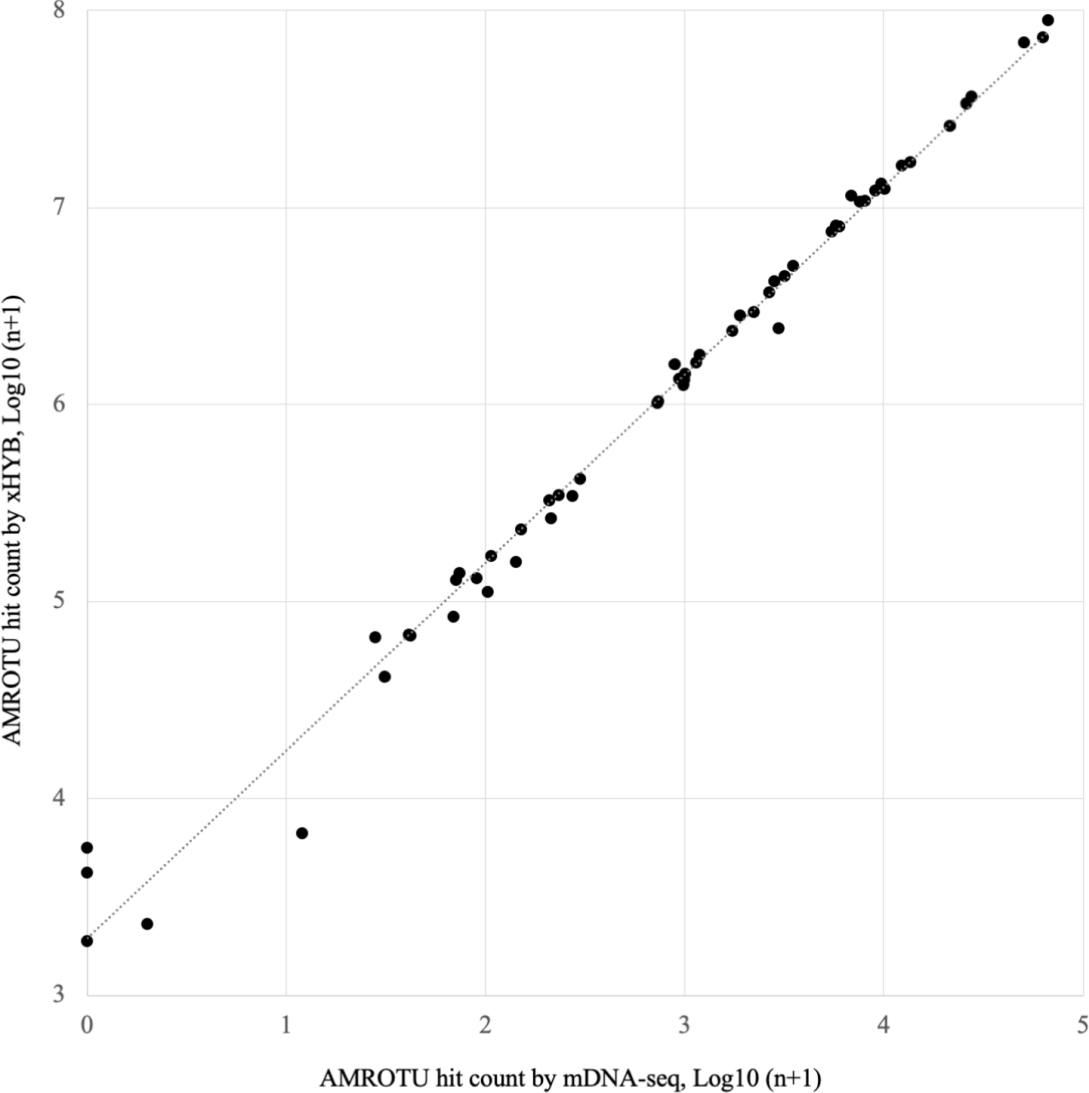
Comparatve ratio of reads per kilobase of gene per million (RPKM) for 328 sampling time points. The AMROTU hit count ratios for xHYB and mDNA-seq were well correlated (*R^2^* = 0.99).

**Fig 2:**
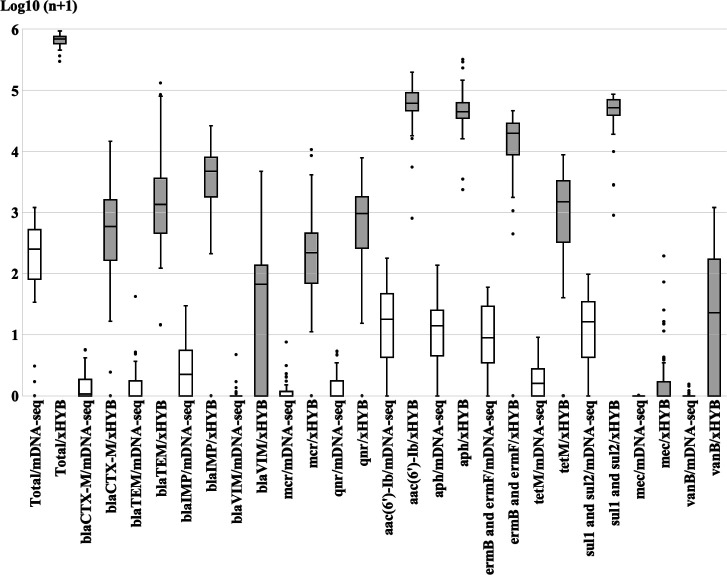
Comparison of reads per kilobase of gene per million (RPKM) value for each antimicrobial resistance gene (ARG) between metagenomic sequencing (mDNA-seq) and hybrid capture (xHYB). In each box and whisker plot, the box marks the interquartile range; the horizontal line across the box shows the median. White box indicates RPKM values of mDNA-seq and glay box indicates that of xHYB.

Overall, xHYB detected significantly more ARGs with sufficient RPKM values (≥1) than mDNA-seq (453, 78, and *P* < 0.05; Table S1). The AMROTU hit count ratios for xHYB and mDNA-seq were well correlated (*R^2^* = 0.99, Fig. 1). The average reads per kilobase per million (RPKM) values for all detected ARGs were 328 by mDNA-seq and 665,225 by xHYB, indicating significantly increased detection by xHYB (3.62 log_10_-fold increase, *P* < 0.05; Fig. 2). The average RPKM values of *bla*_CTX-M_, *bla*_TEM_, *bla*_IMP_, *bla*_VIM_, *mcr*, *qnrS*, *aac(6′)-Ib*, *aph*, *ermB*, *ermF*, *tetM*, *sul1* and *sul2*, *mecA*, *vanA*, and *vanB* were 1, 1, 4, 0, 0, 1, 30, 20, 16, 1, 22, 0, 0, and 0, respectively, for mDNA-seq, and were 1,330, 9,120, 6,173, 224, 777, 1,272, 69,921, 61,927, 18,854, 1,885, 51,788, 6, 0, and 125, respectively, for xHYB (Table 1 and Table S1). Thus, those determined by xHYB were significantly higher than those determined by mDNA-seq (*P* < 0.05; Fig. 2).

**TABLE 1:** Average reads per kilobase of gene per million (RPKM) values of *bla*_CTX-M_, *bla*_TEM_, *bla*_IMP_, *bla*_VIM_, *mcr*, *qnrS*, *aac(6′)-Ib*, *aph*, *ermB* and *ermF*, *tetM*, *sul1* and *sul2*, *mecA*, *vanA,* and *vanB* determined by metagenomic sequencing (mDNA-seq) and the hybrid capture (xHYB)

Gene	Group	AMROTU ID	Gene list	Average RPKM value	
				mDNA-Seq	xHYB
*bla*_CTX-M_	Total			1	1,330
	*bla*_CTX-M-1_	AMROTU_507	*bla*_CTX-M-1,_ *bla*_CTX-M-3,_ *bla*_CTX-M-10,_ *bla*_CTX-M-12,_ *bla*_CTX-M-22,_ *bla*_CTX-M-23,_ *bla*_CTX-M-28,_ *bla*_CTX-M-29,_ *bla*_CTX-M-30,_ *bla*_CTX-M-32,_ *bla*_CTX-M-33,_ *bla*_CTX-M-34,_ *bla*_CTX-M-36,_ *bla*_CTX-M-37,_ *bla*_CTX-M-42,_ *bla*_CTX-M-52,_ *bla*_CTX-M-53,_ *bla*_CTX-M-54,_ *bla*_CTX-M-55,_ *bla*_CTX-M-58,_ *bla*_CTX-M-60,_ *bla*_CTX-M-61,_ *bla*_CTX-M-62,_ *bla*_CTX-M-66,_ *bla*_CTX-M-68,_ *bla*_CTX-M-69,_ *bla*_CTX-M-71,_ *bla*_CTX-M-72,_ *bla*_CTX-M-79,_ *bla*_CTX-M-80,_ *bla*_CTX-M-82,_ *bla*_CTX-M-88,_ *bla*_CTX-M-96,_ *bla*_CTX-M-101,_ *bla*_CTX-M-103,_ *bla*_CTX-M-104,_ *bla*_CTX-M-114,_ *bla*_CTX-M-116,_ *bla*_CTX-M-117,_ *bla*_CTX-M-127,_ *bla*_CTX-M-136,_ *bla*_CTX-M-138,_ *bla*_CTX-M-139,_ *bla*_CTX-M-142,_ *bla*_CTX-M-143,_ *bla*_CTX-M-144,_ *bla*_CTX-M-146,_ *bla*_CTX-M-15,_ *bla*_CTX-M-150,_ *bla*_CTX-M-154,_ *bla*_CTX-M-155,_ *bla*_CTX-M-156,_ *bla*_CTX-M-157,_ *bla*_CTX-M-158,_ *bla*_CTX-M-162,_ *bla*_CTX-M-163,_ *bla*_CTX-M-164,_ *bla*_CTX-M-166,_ *bla*_CTX-M-167,_ *bla*_CTX-M-169,_ *bla*_CTX-M-170,_ *bla*_CTX-M-172,_ *bla*_CTX-M-173,_ *bla*_CTX-M-175,_ *bla*_CTX-M-176,_ *bla*_CTX-M-177,_ *bla*_CTX-M-178,_ *bla*_CTX-M-179,_ *bla*_CTX-M-180,_ *bla*_CTX-M-181,_ *bla*_CTX-M-182,_ *bla*_CTX-M-183,_ *bla*_CTX-M-184,_ *bla*_CTX-M-186,_ *bla*_CTX-M-187,_ *bla*_CTX-M-188,_ *bla*_CTX-M-189,_ *bla*_CTX-M-190,_ *bla*_CTX-M-193,_ *bla*_CTX-M-194,_ *bla*_CTX-M-197,_ *bla*_CTX-M-202,_ *bla*_CTX-M-203,_ *bla*_CTX-M-204,_ *bla*_CTX-M-206,_ *bla*_CTX-M-207,_ *bla*_CTX-M-208,_ *bla*_CTX-M-209,_ *bla*_CTX-M-210,_ *bla*_CTX-M-211,_ *bla*_CTX-M-212,_ *bla*_CTX-M-216,_ *bla*_CTX-M-218,_ *bla*_CTX-M-22,_ *bla*_CTX-M-220,_ *bla*_CTX-M-222,_ *bla*_CTX-M-224,_ *bla*_CTX-M-225,_ *bla*_CTX-M-226,_ *bla*_CTX-M-227,_ *bla*_CTX-M-228,_ *bla*_CTX-M-23,_ *bla*_CTX-M-230,_ *bla*_CTX-M-231,_ *bla*_CTX-M-232,_ *bla*_CTX-M-236,_ *bla*_CTX-M-237,_ *bla*_CTX-M-238,_ *bla*_CTX-M-244,_ *bla*_CTX-M-245,_ *bla*_CTX-M-246,_ *bla*_CTX-M-251_	0	556
	*bla*_CTX-M-2_	AMROTU_539	*bla*_CTX-M-2,_ *bla*_CTX-M-4,_ *bla*_CTX-M-5,_ *bla*_CTX-M-6,_ *bla*_CTX-M-7,_ *bla*_CTX-M-20,_ *bla*_CTX-M-31,_ *bla*_CTX-M-35,_ *bla*_CTX-M-43,_ *bla*_CTX-M-44,_ *bla*_CTX-M-56,_ *bla*_CTX-M-59,_ *bla*_CTX-M-76,_ *bla*_CTX-M-77,_ *bla*_CTX-M-92,_ *bla*_CTX-M-95,_ *bla*_CTX-M-97,_ *bla*_CTX-M-115,_ *bla*_CTX-M-124,_ *bla*_CTX-M-131,_ *bla*_CTX-M-141,_ *bla*_CTX-M-165,_ *bla*_CTX-M-171,_ *bla*_CTX-M-200,_ *bla*_CTX-M-229_	0	5
	*bla*_CTX-M-8_	AMROTU_542	*bla*_CTX-M-8_, *bla*_CTX-M-40_, *bla*_CTX-M-23_	0	3
	*bla*_CTX-M-9_	AMROTU_531	*bla*_CTX-M-9,_ *bla*_CTX-M-13,_ *bla*_CTX-M-14,_ *bla*_CTX-M-14b,_ *bla*_CTX-M-16,_ *bla*_CTX-M-17,_ *bla*_CTX-M-19,_ *bla*_CTX-M-21,_ *bla*_CTX-M-24,_ *bla*_CTX-M-27,_ *bla*_CTX-M-38,_ *bla*_CTX-M-46,_ *bla*_CTX-M-47,_ *bla*_CTX-M-48,_ *bla*_CTX-M-49,_ *bla*_CTX-M-50,_ *bla*_CTX-M-51,_ *bla*_CTX-M-65,_ *bla*_CTX-M-67,_ *bla*_CTX-M-73,_ *bla*_CTX-M-81,_ *bla*_CTX-M-83,_ *bla*_CTX-M-84,_ *bla*_CTX-M-85,_ *bla*_CTX-M-86,_ *bla*_CTX-M-87,_ *bla*_CTX-M-90,_ *bla*_CTX-M-93,_ *bla*_CTX-M-98,_ *bla*_CTX-M-99,_ *bla*_CTX-M-102,_ *bla*_CTX-M-104,_ *bla*_CTX-M-105,_ *bla*_CTX-M-110,_ *bla*_CTX-M-111,_ *bla*_CTX-M-112,_ *bla*_CTX-M-113,_ *bla*_CTX-M-121,_ *bla*_CTX-M-122,_ *bla*_CTX-M-125,_ *bla*_CTX-M-126,_ *bla*_CTX-M-129,_ *bla*_CTX-M-130,_ *bla*_CTX-M-134,_ *bla*_CTX-M-137,_ *bla*_CTX-M-140,_ *bla*_CTX-M-147,_ *bla*_CTX-M-148,_ *bla*_CTX-M-159,_ *bla*_CTX-M-161,_ *bla*_CTX-M-168,_ *bla*_CTX-M-174,_ *bla*_CTX-M-191,_ *bla*_CTX-M-192,_ *bla*_CTX-M-195,_ *bla*_CTX-M-196,_ *bla*_CTX-M-198,_ *bla*_CTX-M-201,_ *bla*_CTX-M-213,_ *bla*_CTX-M-214,_ *bla*_CTX-M-215,_ *bla*_CTX-M-219,_ *bla*_CTX-M-221,_ *bla*_CTX-M-223,_ *bla*_CTX-M-233,_ *bla*_CTX-M-235,_ *bla*_CTX-M-239,_ *bla*_CTX-M-240,_ *bla*_CTX-M-241,_ *bla*_CTX-M-242,_ *bla*_CTX-M-243,_ *bla*_CTX-M-252_	0	765
	*bla*_CTX-M-1/9_ hybrids	AMROTU_540	*bla*_CTX-M-64,_ *bla*_CTX-M-123,_ *bla*_CTX-M-132,_ *bla*_CTX-M-153,_ *bla*_CTX-M-199,_ *bla*_CTX-M-234_	0	1
*bla*_TEM_		AMROTU_584	*bla*_TEM-1,_ *bla*_TEM-1A,_ *bla*_TEM-1C,_ *bla*_TEM-1D,_ *bla*_TEM-2,_ *bla*_TEM-3,_ *bla*_TEM-4,_ *bla*_TEM-5,_ *bla*_TEM-6,_ *bla*_TEM-7,_ *bla*_TEM-8,_ *bla*_TEM-9,_ *bla*_TEM-10,_ *bla*_TEM-11,_ *bla*_TEM-12,_ *bla*_TEM-15,_ *bla*_TEM-16,_ *bla*_TEM-17,_ *bla*_TEM-19,_ *bla*_TEM-20,_ *bla*_TEM-21,_ *bla*_TEM-22,_ *bla*_TEM-24,_ *bla*_TEM-26,_ *bla*_TEM-28,_ *bla*_TEM-29,_ *bla*_TEM-30,_ *bla*_TEM-31,_ *bla*_TEM-32,_ *bla*_TEM-33,_ *bla*_TEM-34,_ *bla*_TEM-35,_ *bla*_TEM-36,_ *bla*_TEM-37,_ *bla*_TEM-39,_ *bla*_TEM-40,_ *bla*_TEM-43,_ *bla*_TEM-45,_ *bla*_TEM-47,_ *bla*_TEM-48,_ *bla*_TEM-49,_ *bla*_TEM-52,_ *bla*_TEM-52B,_ *bla*_TEM-52C,_ *bla*_TEM-53,_ *bla*_TEM-54,_ *bla*_TEM-55,_ *bla*_TEM-57,_ *bla*_TEM-60,_ *bla*_TEM-61,_ *bla*_TEM-63,_ *bla*_TEM-67,_ *bla*_TEM-68,_ *bla*_TEM-70,_ *bla*_TEM-71,_ *bla*_TEM-72,_ *bla*_TEM-76,_ *bla*_TEM-77,_ *bla*_TEM-78,_ *bla*_TEM-79,_ *bla*_TEM-80,_ *bla*_TEM-81,_ *bla*_TEM-82,_ *bla*_TEM-83,_ *bla*_TEM-84,_ *bla*_TEM-85,_ *bla*_TEM-86,_ *bla*_TEM-87,_ *bla*_TEM-88,_ *bla*_TEM-90,_ *bla*_TEM-91,_ *bla*_TEM-92,_ *bla*_TEM-93,_ *bla*_TEM-94,_ *bla*_TEM-95,_ *bla*_TEM-96,_ *bla*_TEM-97,_ *bla*_TEM-98,_ *bla*_TEM-99,_ *bla*_TEM-101,_ *bla*_TEM-102,_ *bla*_TEM-103,_ *bla*_TEM-104,_ *bla*_TEM-105,_ *bla*_TEM-106,_ *bla*_TEM-107,_ *bla*_TEM-108,_ *bla*_TEM-109,_ *bla*_TEM-110,_ *bla*_TEM-111,_ *bla*_TEM-112,_ *bla*_TEM-113,_ *bla*_TEM-114,_ *bla*_TEM-115,_ *bla*_TEM-116,_ *bla*_TEM-120,_ *bla*_TEM-121,_ *bla*_TEM-122,_ *bla*_TEM-123,_ *bla*_TEM-124,_ *bla*_TEM-125,_ *bla*_TEM-126,_ *bla*_TEM-127,_ *bla*_TEM-128,_ *bla*_TEM-129,_ *bla*_TEM-130,_ *bla*_TEM-131,_ *bla*_TEM-132,_ *bla*_TEM-133,_ *bla*_TEM-134,_ *bla*_TEM-135,_ *bla*_TEM-136,_ *bla*_TEM-137,_ *bla*_TEM-138,_ *bla*_TEM-139,_ *bla*_TEM-141,_ *bla*_TEM-142,_ *bla*_TEM-143,_ *bla*_TEM-144,_ *bla*_TEM-145,_ *bla*_TEM-146,_ *bla*_TEM-147,_ *bla*_TEM-148,_ *bla*_TEM-149,_ *bla*_TEM-150,_ *bla*_TEM-151,_ *bla*_TEM-152,_ *bla*_TEM-153,_ *bla*_TEM-154,_ *bla*_TEM-155,_ *bla*_TEM-156,_ *bla*_TEM-157,_ *bla*_TEM-158,_ *bla*_TEM-159,_ *bla*_TEM-160,_ *bla*_TEM-162,_ *bla*_TEM-163,_ *bla*_TEM-164,_ *bla*_TEM-166,_ *bla*_TEM-167,_ *bla*_TEM-168,_ *bla*_TEM-169,_ *bla*_TEM-171,_ *bla*_TEM-176,_ *bla*_TEM-177,_ *bla*_TEM-178,_ *bla*_TEM-181,_ *bla*_TEM-182,_ *bla*_TEM-183,_ *bla*_TEM-184,_ *bla*_TEM-185,_ *bla*_TEM-186,_ *bla*_TEM-187,_ *bla*_TEM-188,_ *bla*_TEM-189,_ *bla*_TEM-190,_ *bla*_TEM-191,_ *bla*_TEM-193,_ *bla*_TEM-194,_ *bla*_TEM-195,_ *bla*_TEM-196,_ *bla*_TEM-197,_ *bla*_TEM-198,_ *bla*_TEM-201,_ *bla*_TEM-205,_ *bla*_TEM-206,_ *bla*_TEM-207,_ *bla*_TEM-208,_ *bla*_TEM-209,_ *bla*_TEM-210,_ *bla*_TEM-211,_ *bla*_TEM-212,_ *bla*_TEM-213,_ *bla*_TEM-214,_ *bla*_TEM-215,_ *bla*_TEM-216,_ *bla*_TEM-217,_ *bla*_TEM-219,_ *bla*_TEM-220,_ *bla*_TEM-224,_ *bla*_TEM-225,_ *bla*_TEM-226,_ *bla*_TEM-227,_ *bla*_TEM-228,_ *bla*_TEM-229,_ *bla*_TEM-230,_ *bla*_TEM-231,_ *bla*_TEM-232,_ *bla*_TEM-233,_ *bla*_TEM-234,_ *bla*_TEM-235,_ *bla*_TEM-236,_ *bla*_TEM-237,_ *bla*_TEM-238,_ *bla*_TEM-239,_ *bla*_TEM-240,_ *bla*_TEM-241,_ *bla*_TEM-242,_ *bla*_TEM-243,_ *bla*_TEM-244,_ *bla*_TEM-245,_ *bla*_TEM-246_	1	9,120
*bla*_IMP_	Total			4	6,173
	*bla*_IMP-1_	AMROTU_873	*bla*_IMP-1,_ *bla*_IMP-3,_ *bla*_IMP-6,_ *bla*_IMP-7,_ *bla*_IMP-10,_ *bla*_IMP-25,_ *bla*_IMP-26,_ *bla*_IMP-30,_ *bla*_IMP-34,_ *bla*_IMP-38,_ *bla*_IMP-40,_ *bla*_IMP-42,_ *bla*_IMP-43,_ *bla*_IMP-51,_ *bla*_IMP-52,_ *bla*_IMP-55,_ *bla*_IMP-59,_ *bla*_IMP-60,_ *bla*_IMP-61,_ *bla*_IMP-66,_ *bla*_IMP-70,_ *bla*_IMP-73,_ *bla*_IMP-76,_ *bla*_IMP-77,_ *bla*_IMP-78,_ *bla*_IMP-79,_ *bla*_IMP-80,_ *bla*_IMP-88,_ *bla*_IMP-89,_ *bla*_IMP-94_	4	6,130
	*bla*_IMP-2_	AMROTU_864	*bla*_IMP-2,_ *bla*_IMP-8,_ *bla*_IMP-13,_ *bla*_IMP-17,_ *bla*_IMP-19,_ *bla*_IMP-20,_ *bla*_IMP-23,_ *bla*_IMP-24,_ *bla*_IMP-_ _33,_ *bla*_IMP-37,_ *bla*_IMP-39,_ *bla*_IMP-69,_ *bla*_IMP-84_	0	6
	*bla*_IMP-5_	AMROTU_887	*bla*_IMP-5,_ *bla*_IMP-9,_ *bla*_IMP-15,_ *bla*_IMP-28,_ *bla*_IMP-29,_ *bla*_IMP-45,_ *bla*_IMP-53,_ *bla*_IMP-62,_ *bla*_IMP-_ _81,_ *bla*_IMP-82,_ *bla*_IMP-85_	0	6
	*bla*_IMP-11_	AMROTU_876	*bla*_IMP-11,_ *bla*_IMP-16,_ *bla*_IMP-21,_ *bla*_IMP-22,_ *bla*_IMP-41,_ *bla*_IMP-44,_ *bla*_IMP-58,_ *bla*_IMP-68,_ *bla*_IMP-74,_ *bla*_IMP-93_	0	16
	*bla*_IMP-31_	AMROTU_878	*bla*_IMP-31_, *bla*_IMP-35_, *bla*_IMP-92_	0	15
*bla*_VIM_		AMROTU_735	*bla_VIM-1,_ bla_VIM-2,_ bla_VIM-3,_ bla_VIM-4,_ bla_VIM-6,_ bla_VIM-8,_ bla_VIM-9,_ bla_VIM-10,_ bla_VIM-11,_ bla_VIM-12,_ bla_VIM-14,_ bla_VIM-15,_ bla_VIM-16,_ bla_VIM-17,_ bla_VIM-18,_ bla_VIM-19,_ bla_VIM-20,_ bla_VIM-23,_ bla_VIM-24,_ bla_VIM-25,_ bla_VIM-26,_ bla_VIM-27,_ bla_VIM-28,_ bla_VIM-29,_ bla_VIM-30,_ bla_VIM-31,_ bla_VIM-32,_ bla_VIM-33,_ bla_VIM-34,_ bla_VIM-35,_ bla_VIM-36,_ bla_VIM-37,_ bla_VIM-38,_ bla_VIM-39,_ bla_VIM-40,_ bla_VIM-41,_ bla_VIM-42,_ bla_VIM-43,_ bla_VIM-44,_ bla_VIM-45,_ bla_VIM-46,_ bla_VIM-48,_ bla_VIM-50,_ bla_VIM-51,_ bla_VIM-52,_ bla_VIM-53,_ bla_VIM-54,_ bla_VIM-55,_ bla_VIM-56,_ bla_VIM-57,_ bla_VIM-58,_ bla_VIM-59,_ bla_VIM-60,_ bla_VIM-62,_ bla_VIM-63,_ bla_VIM-64,_ bla_VIM-65,_ bla_VIM-66,_ bla_VIM-67,_ bla_VIM-68,_ bla_VIM-70,_ bla_VIM-72,_ bla_VIM-73,_ bla_VIM-74,_ bla_VIM-75,_ bla_VIM-76,_ bla_VIM-77,_ bla_VIM-78,_ bla_VIM-79,_ bla_VIM-80_*	0	224
*mcr*	*mcr-3*	AMROTU_76	*mcr-3.1, mcr-3.10, mcr-3.11, mcr-3.12, mcr-3.13, mcr-3.14, mcr-3.15, mcr-3.16, mcr-3.18, mcr-3.19, mcr-3.2, mcr-3.20, mcr-3.21, mcr-3.22, mcr-3.23, mcr-3.24, mcr-3.25, mcr-3.26, mcr-3.27, mcr-3.28, mcr-3.29, mcr-3.3, mcr-3.31, mcr-3.32, mcr-3.33, mcr-3.34, mcr-3.35, mcr-3.36, mcr-3.37, mcr-3.38, mcr-3.39, mcr-3.4, mcr-3.40, mcr-3.41, mcr-3.5, mcr-3.6, mcr-3.7, mcr-3.8, mcr-3.9*	0	62
	*mcr-5*	AMROTU_72	*mcr-5.1*, *mcr-5.2*, *mcr-5.3*, *mcr-5.4*	0	660
	*mcr-7*	AMROTU_83	*mcr-7.1*	0	6
	*mcr-9*	AMROTU_84	*mcr-9*	0	26
	*mcr-10*	AMROTU_82	*mcr-10.1, mcr-10.2, mcr-10.3, mcr-10.4, mcr-10.5*	0	20
*qnrS*	Total			1	1,272
		AMROTU_955	*qnrS2, qnrS6*	0	704
		AMROTU_954	*qnrS1, qnrS3, qnrS4, qnrS5, qnrS7, qnrS8, qnrS9, qnrS10, qnrS11, qnrS12, qnrS13, qnrS14, qnrS15*	0	568
*aac(6')-Ib*		AMROTU_1012	*aac(6')-Ib, aac(6')-Ib', aac(6')-Ib3, aac(6')-Ib4, aac(6')-Ib11, aac(6')-Ib-cr, aac(6')-Ib-cr3, aac(6')-Ib-cr4, aac(6')-Ib-cr5, aac(6')-Ib-cr6, aac(6')-Ib-cr7, aac(6')-Ib-cr8, aac(6')-Ib-cr9, aac(6')-Ib-cr10, aac(6')-Ib-cr11, aac(6')Ib-cr, aac(6')-Ib-Hangzhou*	30	69,921
*aph*	Total			20	61,927
		AMROTU_714	*aph(3')-Ia*	1	20,569
		AMROTU_658	*aph (6)-Id*	9	19,310
		AMROTU_674	*aph(3'')-Ib*	9	18,272
		AMROTU_775	*aph(3')-IIIa*	1	2,692
		AMROTU_813	*aph(3')-VI, aph(3')-VIa, aph(3')-VIb*	0	577
		AMROTU_716	*aph(3')-Ib*	0	137
		AMROTU_776	*aph(3')-IIa*	0	94
		AMROTU_737	*aph(3')-IIb*	0	79
		AMROTU_739	*aph(3')-XV*	0	48
		AMROTU_638	*aph (6)-Ic*	0	37
		AMROTU_478	*aph(2'')-If*	0	37
		AMROTU_461	*aph(2'')-IIa*	0	33
		AMROTU_415	*aph(2'')-Ig*	0	27
		AMROTU_449	*aph(2'')-Ie, aph(2'')-IVa*	0	7
		AMROTU_414	*aph(2'')-IIIa*	0	4
		AMROTU_854	*aph(3')-VIIa*	0	2
		AMROTU_747	*aph(3')-IIc*	0	2
*ermB and ermF*	Total			16	18,854
	*ermB*	AMROTU_764	*ermB*	5	6,798
	*ermF*	AMROTU_844	*ermF*	10	12,056
*tetM*		AMROTU_44	*tetM, tetS/M*	1	1,885
*sul1 and sul2*	Total			22	51,788
	*sul1*	AMROTU_525	*sul1*	17	42,156
	*sul2*	AMROTU_622	*sul2*	4	9,632
*mecA*		AMROTU_32	*mecA, mecA2*	0	6
*vanB*		AMROTU_328	*vanB*	0	125

For CTX-M-type ESBL genes, the average xHYB RPKM values of *bla*_CTX-M-1_ group, including *bla*_CTX-M-15_, and *bla*_CTX-M-9_ group, including *bla*_CTX-M-14_ and *bla*_CTX-M-27_, mainly produced by ESBL-producing *Enterobacterales* in Japan (6), were significantly higher than those of *bla*_CTX-M-2_, *bla*_CTX-M-8_, and *bla*_CTX-M-1/9_ hybrid groups (556 and 765 vs 5, 3, and 1, respectively, *P* < 0.05; Table 1). *bla*_IMP_ and *bla*_VIM_ were the only MBL-type carbapenemase genes detected in the wastewater; *bla*_KPC_ and *bla*_NDM-1_ were not found. The average xHYB RPKM values of *bla*_IMP-1_ group, including *bla*_IMP-1_ and *bla*_IMP-6_, mainly produced by carbapenemase-producing *Enterobacterales* in Japan (7), were significantly higher than those of *bla*_IMP-2_, *bla*_IMP-5_, *bla*_IMP-11_, and *bla*_IMP-31_ groups and *bla*_VIM_ (6,130 vs 6, 6, 16, 15, and 224, respectively, and *P* < 0.05). For the *mcr* genes, the average xHYB RPKM value of *mcr-5* group was significantly higher than those of *mcr-3*, *mcr-7*, *mcr-9*, and *mcr-10* groups (660 vs 62, 6, 26, and 20, respectively, *P* < 0.05). None of these genes were detected by mDNA-seq, except *bla*_IMP-1_ group, with an RPKM value of 4.

### Comparison between ARGs in hospital effluent and clinical ARB isolates

**Fig 3:**
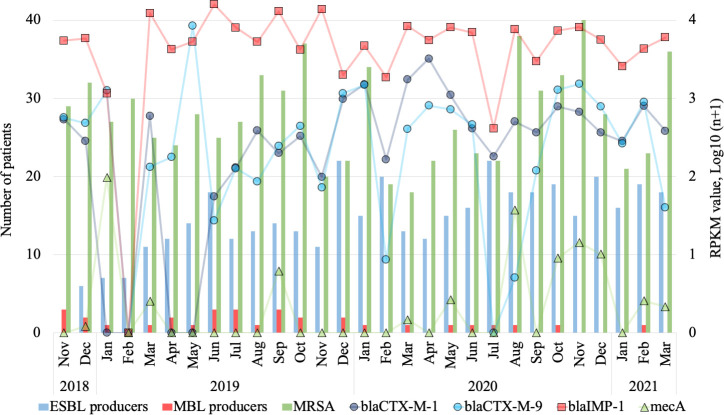
The monthly average number of patients with extended-spectrum β-lactamase (ESBL)-producing and metallo-β-lactamase (MBL)-producing bacteria, and MRSA, and the monthly reads per kilobase of gene per million (RPKM) values of *bla*_CTX-M-1_, *bla*_CTX-M-2_, *bla*_IMP-1_, and *mecA*. The left axis showed the number of patients, and the right showed the RPKM value, Log10(n+1). Blue, red, and green bars indicate the number of patients with ESBL and MBL-producers, and MRSA, respectively. Purple and blue circle, red square, and green triangle indicate RPKM values of *bla*_CTX-M-1_, *bla*_CTX-M-2_, *bla*_IMP-1_, and *mecA*, respectively.

ESBL-producing *E. coli*, *K. pneumoniae*, *K. oxytoca*, and *P. mirabilis* were detected, averaging 10, 3, 1, and 1 patient per month, and the monthly average was significantly higher in 2020 than in 2019 (17 vs 13 patients/month and *P* < 0.05) (Fig. 3). In parallel with this increase, the average monthly RPKM values of *bla*_CTX-M-1_ group was significantly higher in 2020 than in 2019 (921 vs 232, and *P* < 0.05), while the average RPKM values of *bla*_CTX-M-9_ group in 2019 and 2020 were similar (1,016 vs 636 per month and *P* ≥ 0.05).

Significantly fewer patients carried metallo-β-lactamase (MBL) producers, including *E. coli*, *K. pneumoniae*, *Enterobacter cloacae*, *Pseudomonas aeruginosa*, and *Acinetobacter* spp., than carried ESBL producers (1 vs 14 patients per month and *P* < 0.05) (Fig. 3). The mean RPKM value of *bla*_IMP_ in effluent was significantly higher than that of *bla*_CTX-M_ (6,163 vs 1,327 per month and *P* < 0.05). Significantly more patients carried methicillin-resistant *Staphylococcus aureus* (MRSA) than carried ESBL producers (28 vs 14 patients per month and *P* < 0.05). The average RPKM value of *mecA* was significantly lower than that of *bla*_CTX-M_ (6, 1,327 per month, and *P* < 0.05). VRE was not clinically isolated during the study period, and although *vanA* was not detected in the hospital effluent, *vanB* was detected, with an RPKM value of 126 per month.

## DISCUSSION

This study demonstrated that xHYB could detect clinically important ARGs, including *bla*_CTX-M_ and *bla*_IMP_, most of which were not well detected by conventional mDNA-seq and can effectively depict the ARG profile in hospital effluent. ARGs frequently detected among clinical ARB in Japan, including *bla*_CTX-M-1_, *bla*_CTX-M-9_, and *bla*_IMP-1_ genes, were not detected by mDNA-seq. However, xHYB revealed that they were more abundant in the hospital effluent than other ARGs of the same type. An increase in the frequency of ESBL-producing isolates was observed, paralleled by an increased abundance of *bla*_CTX-M-1_ genes in the effluent.

Although most ESBLs in clinical isolates in Japan are *bla*_CTX-M_ (8, 9), in this study, the RPKM value of *bla*_TEM_ was higher than that of *bla*_CTX-M_. Although all previously identified *bla*_CTX-M_ genes confer an ESBL phenotype, the operational taxonomic unit (OTU) of *bla*_TEM_ in the AMROTU database in the present study contains narrow-spectrum *bla*_TEM_ genes, including *bla*_TEM-1_ and *bla*_TEM-2_, which are widely distributed among gram-negative rods in water environments (10). Among the *bla*_CTX-M_ genes detected in the hospital effluent, *bla*_CTX-M-9_ was most abundant, with a total RPKM of 765, followed by *bla*_CTX-M-1_, with 556, accounting for 99% of the *bla*_CTX-M_ genes. Among the major ESBL producers isolated, *E. coli* was most abundant (71%). These results were consistent with previous clinical epidemiological studies of ESBL producers in Japan (6, 9). The recent rapid increase in the prevalence of *bla*_CTX-M_, the largest group of ESBLs, has become a major public health concern worldwide (6). In Japan, the prevalence of *bla*_CTX-M-27_ producers (*bla*_CTX-M-9_ group) is rapidly increasing in addition to the globally prevalent *bla*_CTX-M-15_ (*bla*_CTX-M-1_ group), replacing *bla*_CTX-M-14_ (*bla*_CTX-M-9_ group) (6, 11). In the present study, the increased abundance of *bla*_CTX-M-1_ group in hospital effluent paralleled the frequency of ESBL-producing bacterial isolates.

The most frequent and clinically important carbapenemase genes are *bla*_NDM-1_, *bla*_VIM_, *bla*_KPC_, and *bla*_IMP_ (12). None of these genes was detected by mDNA-seq, whereas *bla*_VIM_ and *bla*_IMP_ were detected by xHYB. *bla*_IMP-1_ group and *bla*_VIM_, with a total RPKM of 6,130 and 224, accounted for 96% and 4% of the carbapenemase genes in the hospital effluent, respectively, which is consistent with previous clinical epidemiological studies showing that the carbapenemases detected in Japan were mostly *bla*_IMP-1_ group genes, whereas *bla*_VIM_, *bla*_NDM-1_, and *bla*_KPC_ were rarely detected (7, 13). In recent years, there has been a reported increase in the prevalence of clinical isolates of *bla*_VIM_-producing *P. aeruginosa* in Japan (14); however, the origin of the *bla*_VIM_ in this study remains uncertain. The RPKM value of *bla*_IMP_ in this study was higher than that of *bla*_CTX-M_, while fewer patients had MBL producers than ESBL producers. This finding may be attributed to the fact that many MBL producers, such as *P. aeruginosa*, *Acinetobacter* spp., and *Aeromonas* spp., are opportunistic pathogens with wide environmental distribution, including in wastewater, and are not part of the human flora, unlike *Enterobacterales*, to which ESBL producers mainly belong (15, 16). Contamination of wastewater systems with ARB indicates a potential transmission risk to inpatients (17). Monitoring sewage for MBL genes in regions where MBL-type carbapenemases are endemic may provide insight into the extent of hospital water contamination by MBL-producing bacteria.

Among the colistin-resistance genes, *mcr-1*, which is the predominant *mcr* gene worldwide (18), was not detected. xHYB primarily detected *mcr-5*, as well as *mcr-3* and *mcr-9*, although at low RPKM values. Although rare in Japan, clinical isolates carrying *mcr-5* and *mcr-9* were detected in a nationwide survey (19). Since strains carrying *mcr-5* and *mcr-9* may be susceptible to low colistin concentrations (19), monitoring their dissemination within hospitals using culture-independent methods could help prevent their spread.

xHYB also detected resistance genes for quinolone (*qnr*), aminoglycosides (*aac(6′)-Ib* and *aph*), macrolides (*ermB* and *ermF*), and sulfonamides (*sul1*) at significantly higher RPKM values than those obtained using mDNA-seq. The most significant quinolone resistance mechanism in *Enterobacterales* involves the accumulation of mutations in the quinolone resistance-determining regions of DNA gyrase and topoisomerase IV, which cannot be detected by either mDNA-seq or xHYB (20, 21); however, the widespread emergence of plasmid-mediated quinolone resistance (PMQR) genes, including *aac(6′)-Ib-cr*, a variant of *aac(6′)-Ib*, and *qnr*, has become a worldwide concern (22). The abundance of PMQR and macrolide resistance genes in sewage reflects the respective clinical use of quinolones and macrolides (21). The presence of *sul1* in wastewater strongly correlates with anthropogenic inputs and is associated with horizontal gene transfer (HGT) (23). Therefore, monitoring effluent ARGs may help assess the extent of hospital dissemination and estimate antimicrobial usage and the rate and extent of HGT.

Although we infrequently detected *mecA* in hospital effluent, the number of patients with MRSA exceeded the number of patients carrying ESBL or MBL producers. *S. aureus* is a commensal bacterium colonizing skin and nasal passages that is less prevalent in gastrointestinal and genitourinary tracts than *Enterobacterales* (24). Thus, surveying resistance genes in sewage may be insufficient for monitoring inpatient spread of pathogens not typically found in gastrointestinal or genitourinary tracts.

The study found no VRE clinical isolates, and *vanA* was not detected in the hospital effluent; however, *vanB* was detected by the xHYB method. VRE strains carrying *vanA* are resistant to vancomycin and teicoplanin, while strains carrying *vanB* are resistant to vancomycin and susceptible to teicoplanin (25), rendering them undetectable through conventional drug-susceptibility testing methods. As such, they may go clinically unrecognized.

This study has several limitations. First, the xHYB-detectable ARGs are limited to those in the QIAseq xHYB AMR Panel, and the xHYB method is unable to distinguish the number of reads for variants within the same group, such as *bl*a_CTX-M-14_ and *bla*_CTX-M-27_, *bla*_IMP-1_ and *bla*_IMP-6_, and *aac(6′)-Ib* and *aac(6′)-Ib-cr*. However, the performance of xHYB seems sufficient for comprehensive detection of ARGs in hospital effluent, since the 2,786 target ARGs cover 93.1% of those registered in the NCBI/ResFinder database, and those remaining are not worldwide concerns. Second, this study did not evaluate the residual antimicrobials in hospital effluent that may influence the abundance of AMR in wastewater (1, 21). Third, as the number of inpatients with ARB was analysed retrospectively, it may be an underestimate since not all patients underwent available culture tests and only prevalent ARB were targeted. Fourth, the culture-independent methods including mDNA-seq and xHYB are unable to determine the specific bacterial strain responsible for the detected ARGs. Nonetheless, correlations were observed between the numbers of inpatients with ARB and the ARG RPKM values in hospital effluent over time. In conclusion, this study demonstrates that xHYB appropriately monitors ARGs in hospital effluent for sensitive identification of nosocomial AMR dissemination. ARG surveillance in hospital effluent using the highly sensitive and specific xHYB method could also improve our understanding of the emergence and spread of AMR within a hospital.

## MATERIALS AND METHODS

### Sample collection

Effluent samples were collected twice a month from November 2018 to May 2021 from a sewer pipe connected to inpatient buildings at a university hospital with 1,200 beds and approximately 1,000 new admissions per month. The average discharge from a hospital ward is 250–300 m^3^/day. Hospital wastewater samples were collected into 500 mL sterile tubes and transported to the laboratory on ice. The samples were stored at −80℃ until analysis. To collect organisms larger than bacteria, the water samples were passed through a TPP Rapid Filtermax Vacuum Filtration system (Trasadingen, Switzerland) in bottles fitted with 49 cm^2^ polyethersulfone 0.2 µm membranes. The membranes were removed from the bottles and stored at −30℃ until DNA extraction. A portion (~1/4) of the collected membranes was cut into small pieces and placed into ZR-96 Bashing Bead lysis tubes (0.1 and 0.5 mm; Zymo Inc., Irvine, CA, USA). Roche bacterial lysis buffer (800 µL) was added to the bead tube, frozen at −30℃, and then thawed at 23℃. The tube was subjected to bead-beating (1,500 rpm for 10 min) using a GenoGrinder 2010 (SPEX SamplePrep, Metuchen, NJ, USA). After centrifugation (8,000× *g* for 3 min), 400 µL of the supernatant was collected. The DNA in the supernatant was purified using the Quick-DNA Fecal/Soil Microbe Kit (Zymo Research, Irvine, CA, USA). The DNA concentration and purity were measured using the Qubit DNA HS kit (Thermo Fisher Scientific, Waltham, MA, USA).

### mDNA-seq analysis of water samples

mDNA-seq libraries were prepared using the QIAseq FX DNA library kit (Qiagen, Hilden, Germany) with an index for each sample, followed by short-read sequencing using the NexSeq 500 platform (2 × 150-mer paired-end) (Illumina, San Diego, CA, USA). Adapters and low-quality sequences were trimmed using Sickle version 1.33 (https://github.com/najoshi/sickle) with the following parameters: average quality threshold “-q 20” and minimum length threshold “-l 40” Subsequently, mDNA-seq analysis was performed using clean reads for homology searches without *de novo* assembly.

### Multiplex hybrid capture of targeted ARGs

mDNA-seq libraries were pooled for xHYB-targeted enrichment using the QIAseq xHYB AMR panel kit (Qiagen), which includes 2,786 ARGs, according to the manufacturer’s protocol. The dried libraries and denatured xHYB biotinylated probe panel were mixed and incubated at 70℃ for 18 h for hybridization. The hybridized libraries were captured using streptavidin-coated beads and washed to remove unbound library fragments. The captured DNA libraries were enriched with 20 PCR cycles, followed by Illumina NextSeq 2000 sequencing using NextSeq 1000/2000 P2 Reagent (2 × 150-mer paired-end) (Illumina).

### Resistome analysis

In subsequent analyses, mDNA-seq was performed using clean reads for homology searches without *de novo* assembly. Before resistome analysis, an ARG database was constructed from the National Center for Biotechnology Information (NCBI) Antimicrobial Resistance Reference Gene Database (BioProject ID: PRJNA313047) and ResFinder (https://bitbucket.org/genomicepidemiology/resfinder_db/src/master/) (26) using Makeblastdb in basic local alignment search tool (BLAST+). The OTUs in the ARG database, including 1,272 ARGs (AMROTU version 2022-04-11; Table S2), were created by clustering at ≥90% sequence identity and ≥80% coverage using Vsearch version 2.10.4 (26). The mDNA-seq reads were searched using mega-BLAST (e-value threshold: 1E-20; identity threshold: 95%) against the custom ARG database. The detected genes were summarized for each ARG OTU. For normalization, the RPKM values were calculated using the following formula:


$$RPKM=\frac{number\;of\;detected\;reads\;against\;OTUs}{average\;length\;of\;detected\;OTUs\;(bp)\times total\;number\;of\;trimmed\;reads}\times 10^{9}$$

Table S1 shows the xHYB and mDNA-seq RPKM values of the ARGs in AMROTU. We also compared ARGs with high environmental fitness commonly associated with mobile genetic elements, as suggested by Berendonk et al.: *bla*_CTX-M_, *bla*_TEM_, *bla*_NDM-1_, *bla*_VIM_, *bla*_KPC_, *qnrS*, *aac-(6’)-Ib*, *aph*, *ermB*, *ermF*, *tetM*, *sul1*, *sul2*, *vanA*, and *mecA* (27). *bla*_IMP_, the most prevalent MBL gene in clinical isolates in Japan (28); *mcr*, which confers resistance to colistin, a last-resort antimicrobial (29); and *vanB*, found in most clinically important VRE like *vanA* (30), were also considered.

### Hospital microbiology database

From the hospital microbiology database, we collected monthly data on clinical specimens of ceftriaxone-resistant ESBL-producing bacteria (*Escherichia coli*, *Klebsiella pneumoniae*, *Klebsiella oxytoca*, and *Proteus mirabilis*) (8), MBL-producing gram-negative bacteria, MRSA, and VRE. Species identity and antimicrobial susceptibility were determined using the Vitek-2 system (Siemens Healthcare Diagnostics Japan, Tokyo, Japan). MBL production was detected in gram-negative bacteria resistant to both ceftazidime and sulbactam-cefoperazone by the sodium mercaptoacetic acid disk test (31). All specimen types were included (blood, urine, fecal, other fluid and tissue, and indwelling plastic); duplicate specimens from the same patient collected within a month were excluded. The monthly number of patients with ARB isolates was compared to the monthly average RPKM of *bla*_CTX-M_, *bla*_IMP_, *mecA*, *vanA*, and *vanB*.

### Statistical analysis

Fisher’s exact test was used to compare categorical variables. Welch’s *t*-test and Tukey’s honestly significant difference test were used to compare continuous variables between two groups and more than two groups, respectively. All analyses were performed using JMP Pro 16 (SAS Institute Japan, Tokyo, Japan). A *P* value <0.05 was considered statistically significant.

## SUPPLEMENTAL MATERIAL

TABLE S1 msphere.00100-23-s0001.xlsx Comparison of reads per kilobase of gene per million (RPKM) values for each AMROTUs between metagenomic sequencing (mDNA-seq) and the hybrid capture (xHYB). RPKM values were calculated using the following formula for normalization: RPKM = number of detected reads against OTUs/[average gene length of detected OTUs (bp) x the total number of trimmed reads] x 10^9^.

TABLE S2 msphere.00100-23-s0002.xlsx Operational taxonomic units (OTUs) in the antimicrobial resistance gene (ARG) database (AMROTU ver. 2022-04-11). This database was constructed from the databases, the National Center for Biotechnology Information (NCBI) Antimicrobial Resistance Reference Gene Database (BioProject ID: PRJNA313047) and ResFinder, https://bitbucket.org/genomicepidemiology/resfinder_db/src/master/), using Makeblastdb in basic local alignment search tool (BLAST+). The AMROTUs were created by clustering at ≥90% sequence identity and ≥80% coverage using Vsearch version 2.10.4.

ASM does not own the copyrights to Supplemental Material that may be linked to, or accessed through, an article. The authors have granted ASM a non-exclusive, world-wide license to publish the Supplemental Material files. Please contact the corresponding author directly for reuse.
